# Commissioning and Evaluation of an Electronic Portal Imaging Device-Based In-Vivo Dosimetry Software

**DOI:** 10.7759/cureus.2139

**Published:** 2018-02-02

**Authors:** Mareike Held, Joey Cheung, Angelica Perez Andujar, François Husson, Olivier Morin

**Affiliations:** 1 Radiation Oncology, University of California San Francisco; 2 Dosisoft, N/A

**Keywords:** external beam radiotherapy, in-vivo dosimetry, epid, quality assurance, finite tissue maximum ratio (ftmr)

## Abstract

This study reports on our experience with the in-vivo dose verification software, EPIgray® (DOSIsoft, Cachan, France). After the initial commissioning process, clinical experiments on phantom treatments were evaluated to assess the level of accuracy of the electronic portal imaging device (EPID) based in-vivo dose verification.

EPIgray was commissioned based on the company’s instructions. This involved ion chamber measurements and portal imaging of solid water blocks of various thicknesses between 5 and 35 cm. Field sizes varied between 2 x 2 cm^2^ and 20 x 20 cm^2^. The determined conversion factors were adjusted through an additional iterative process using treatment planning system calculations. Subsequently, evaluation was performed using treatment plans of single and opposed beams, as well as intensity modulated radiotherapy (IMRT) plans, based on recommendations from the task group report TG-119 to test for dose reconstruction accuracy. All tests were performed using blocks of solid water slabs as a phantom.

For single square fields, the dose at isocenter was reconstructed within 3% accuracy in EPIgray compared to the treatment planning system dose. Similarly, the relative deviation of the total dose was accurately reconstructed within 3% for all IMRT plans with points placed inside a high-dose region near the isocenter. Predictions became less accurate than < 5% when the evaluation point was outside the treatment target. Dose at points 5 cm or more away from the isocenter or within an avoidance structure was reconstructed less reliably.

EPIgray formalism accuracy is adequate for an efficient error detection system with verifications performed in high-dose volumes. It provides immediate intra-fractional feedback on the delivery of treatment plans without affecting the treatment beam. Besides the EPID, no additional hardware is required. The software evaluates local point dose measurements to verify treatment plan delivery and patient positioning within 5% accuracy, depending on the placement of evaluation points.

## Introduction

Electronic portal imaging has been established since the 1950s; nevertheless, modern electronic portal imaging devices (EPIDs), which led to a wider spread of commercialization of camera-based systems, were not introduced until the 1980s [1-2]. In radiation oncology, their main purpose was to facilitate patient setup on the treatment machine and to monitor the patient alignment during treatment. Compared to portal films, using EPIDs saved time; however, image quality was considered to be inferior [3]. Based on a survey conducted by the American Association of Physicists in Medicine (AAPM) Radiation Therapy Committee Task Group 58, EPID technology was “underutilized in the U.S.” and was “not used to produce the intended clinical benefit” when their TG 58 Report was published in 2001 [3]. Since then, extensive research has focused on amorphous silicon (aSi) detectors, which led to Varian Medical Systems, Elekta Oncology Systems, and Siemens Medical Systems each integrating EPIDs on their linear accelerators with indirect-detection, active matrix, flat-panels.

Today, EPIDs are used as a standard tool for patient alignment before treatment [2]. Furthermore, flat-panels may be used for machine quality assurance (QA) and in-air pre-treatment intensity-modulated radiotherapy (IMRT) verification [4-10]. The latter aims to reconstruct the entire dose distribution in space as a method of patient-specific pre-treatment plan QA.

Another application is the use of EPIDs for in-vivo dosimetry (IVD) by measuring the transit dose through the patient [2, 11-19]. This approach aims to document the dose delivered to the patient during each fraction and to make sure that this dose is within the reasonable agreement of the planned dose. Due to regulatory obligations, this practice is more common in European clinics. Still, Mijnheer et al. conclude in their 20/20 vision paper that the potential use of IVD has to impact clinical outcome [18]. They recommend that IVD in external beam radiotherapy (EBRT) should be used in each radiotherapy center, in addition to other QA tools. A limited availability of commercial software and the lack of standardization of metrics has made it difficult to follow this recommendation. A number of detectors have been characterized in their dosimetric performance [20-21]. 

This study reports on the commissioning and evaluation of a commercially available software product that uses the EPID for IVD during EBRT. EPIgray (DOSIsoft, Cachan, France) is based on measurements of the finite tissue maximum ratio (fTMR), which is introduced below. Here, we provide levels of accuracy for this fTMR-based IVD software for treatments of simple three dimensional (3D) conformal fields and the different complexities of IMRT plans delivered to homogeneous phantoms. Celi et al. published on their experience with EPIgray for clinical patient plans, which prompts for a comparison of the results in the discussion [22]. 

## Materials and methods

EPIgray software

**Figure 1:**
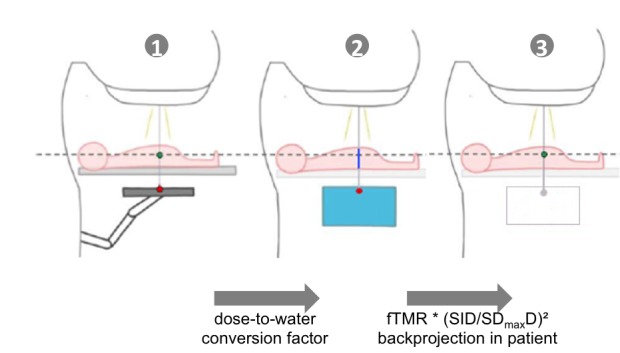
Sketch to explain the conversion from the portal image pixel value to dose inside the patient *Step 1 to 2:* conversion of portal image pixel value into a dose-to-water value at EPID level and depth of D_max_, according to the calibration factor (Gy/EPID_signal) measured in reference conditions (field size, absorber thickness) and used with normalized conversion factors established from a set of different field sizes and absorber thicknesses. *Step 2 to 3:* projecting the dose at D_max_ into the patient by using the fTMR ratio (field size, absorber thickness, air gap), and inverse square law between the imager (source-to-imager distance - SID) and the point located at D_max_ in the patient (source-to-D_max_ distance - SD_max_). For other points of interest, the distance and the depth of the point are used with the appropriate inverse square law and an additional TMR ratio. fTMR: finite tissue maximum ratio; TMR: tissue maximum ratio

Ricketts et al. described the EPIgray formalism. EPIgray uses the finite tissue maximum ratio fTMR, which is the ratio between two doses measured in a phantom at depth D_max_. One dose is measured under the presence of an absorber (here: solid water) with finite dimensions inside the beam path between the source and the detector, while the other is measured under the same conditions but without the absorber [16]. This formalism allows reconstructing the dose in the patient from the dose measured at EPID level in transit conditions. Figure 1 illustrates how the EPID signal is converted to dose at a particular point inside the patient.

**Figure 2:**
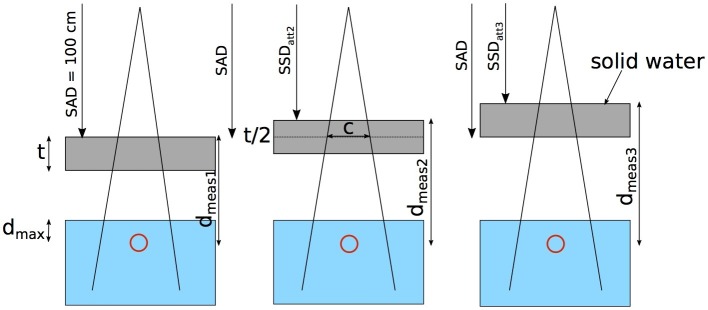
Setup to measure the conversion factors for different SSDatt using solid water as an absorber The red circle indicates the location of the ion chamber in virtual water at D_max_. SAD: source axis distance; SSD_att_: source-to-attenuator-surface distance; D_meas_: attenuator-surface to detector distance

Additionally, a table of conversion factors (FC) is created based on measurements with different field sizes and absorber thicknesses, similar to work by François et al. [17]. These values are used to convert the portal image signal into dose-to-water at the EPID level and depth of D_max_, which is shown in Steps 1 to 2 in Figure 2. Then, the dose is projected into the patient by using the fTMR and the inverse square law between the source-to-imager distance and the source-to-D_max_ distance.

Software Commissioning

The software EPIgray was commissioned on our clinical TrueBeam® STx (Varian, Palo Alto, CA) following the company’s instructions (EPIgray Practical Guide, Edition 2). This required the modeling of the treatment beam and the EPID system in the “EPIgray library” module.

**Figure 3:**
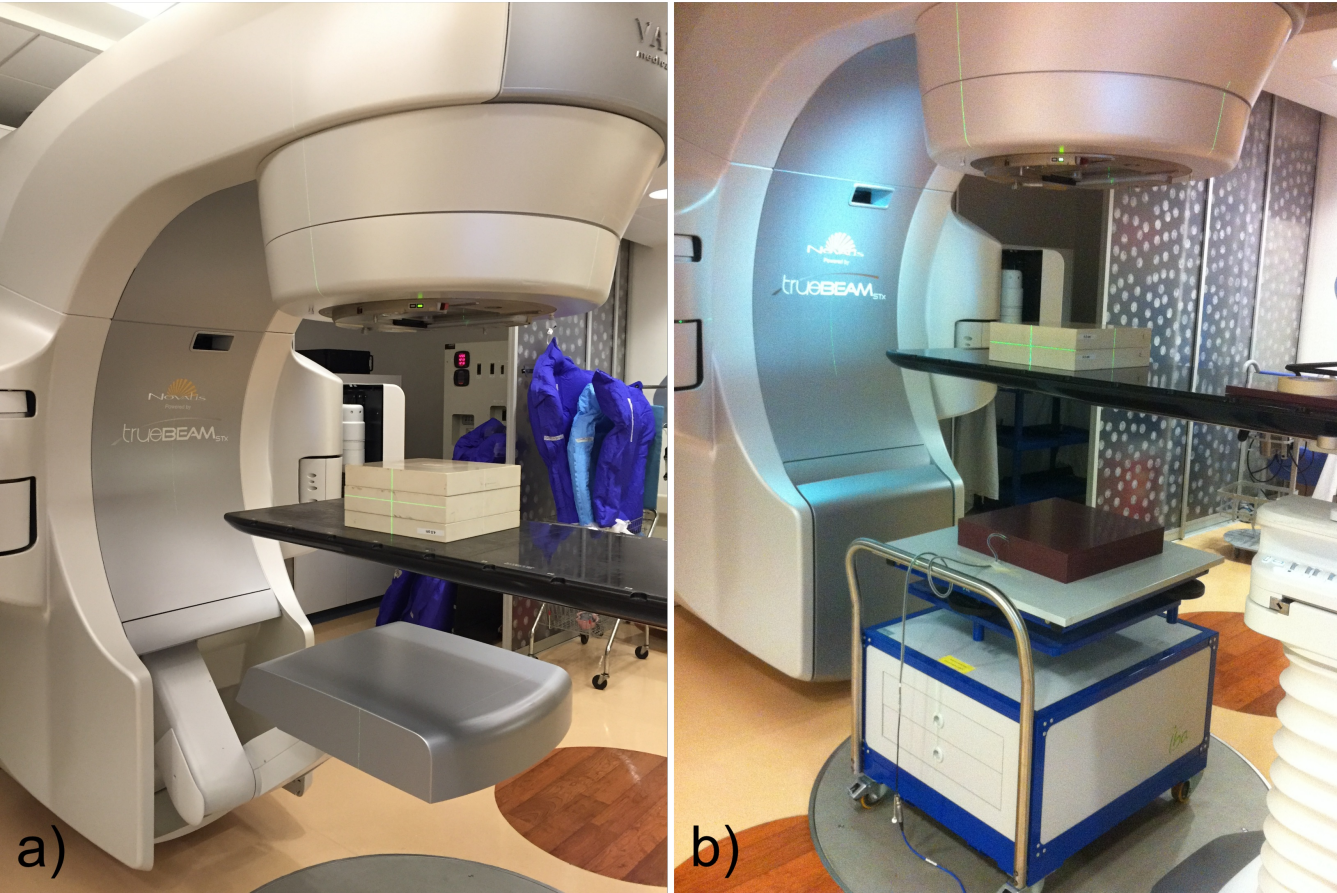
Measurement setup a) Dose measured in solid water. b) Portal image captured with the electronic portal imaging device (EPID).

First, the EPID was calibrated according to the EPID commissioning procedure provided by the vendor, which included the application of the dark field, flood field, and pixel map corrections. This was performed using the TrueBeam Imaging System Calibration workflow. To calibrate the megavoltage (MV) dosimetry mode, diagonal beam profiles are imported as described in the Varian User Guide. Subsequently, the EPIgray calibration and conversion factors were defined. This was performed through a series of measurements, which compared the measured values of the EPID to the dose measured in virtual water. The dose was measured for a range of different setups, which included changing the field size, the thickness of the absorber - blocks of solid water (Radiation Products Design, Inc., Albertville, MN) - between the beam and detector, and the source-to-attenuator-surface distance (SSD_att_). To record the dose per field, the dose was integrated over the time of delivery per field. Figure 2 is a sketch of the measurement setup using the ion chamber placed in virtual water. The SSD_att_ was varied by increments half of the attenuator thickness (t). Each measurement was acquired for field sizes between 2 cm x 2 cm and 24 cm x 24 cm at source-axial-distance (SAD) for 6 MV beam energy. The maximum field size is limited by the EPID size when placed at 150 cm source-to-imager distance (SID). All dose measurements used a cc04 ion chamber (IBA Dosimetry, Schwarzenbruck, Germany) inserted into a 40 x 40 cm^2^ large and 5 cm thick slab of virtual water at the depth of D_max_. Additional details are presented in the EPIgray Practical Guide (EPIgray Practical Guide, Edition 2) that is provided with the software. Figure 3 shows a photo of the commissioning setups. In our case, the conversion factors were adjusted using calculated dose values at isocenter for different solid water blocks from the treatment planning system (TPS) Eclipse™ (Varian Medical Systems, Inc., Palo Alto, CA). This was necessary to correct for systematic differences between the measurement conditions and the TPS conditions.

TPS Correction for Conversion Factors

The conversion factors were adjusted through an iterative measurement process. Therefore, in the TPS, the dose of square treatment fields was calculated at the field isocenter inside solid water. Treatment fields with field sizes of 2 x 2, 4 x 4, 8 x 8, 10 x 10, 15 x 15, and 20 x 20 cm^2^ were planned at 100 cm SAD on solid water phantoms of varying thicknesses (5, 10, 20, and 30 cm). The calculated dose at isocenter was recorded for each phantom thickness and each field size.

The treatment fields were delivered as planned with the flat panel extended at 150 cm SID to record the portal image. The raw image value calibrated units (CU) at the center of each portal image was read out using the Digital Imaging and Communications in Medicine (DICOM) viewer software OsiriX (Pixmeo, Bernex, Switzerland).

The conversion factors FC mentioned above were calculated by

$$FC = \frac{D_{TPS}}{D_{calib} \times CU} \times \left(\frac{SID}{SAD}\right)^2 \times \frac{fTMR_{(phantom\_thickness)}}{TMR_{(isocenter\_depth)}},$$

where $D_{TPS}$ is the dose calculated by the treatment planning system at isocenter, $D_{calib}$ is the calibration dose at D_max_ for 100 monitor units (MU) delivered by a 10 x 10 cm^2^ field size and a 100 cm SAD setup, $CU$ is the calibration unit read out from the portal image, and $\left(\frac{SID}{SAD}\right)^2$ is the inverse square distance correction from the source-to-imager distance to isocenter. For a conventional linac setup, $D_{calib}$ equals 1. The term $\frac{fTMR}{TMR}$ is included to calculate the dose at isocenter for various phantom thicknesses.

Phantom in vivo measurements

Single Field Plans

During the first part of this study, EPIgray was used to validate the dose of individual square fields. The absorber material between the linac head and EPID was a stack of solid water with a size of 30 x 30 cm2 and a thickness of 5 cm, 15 cm, and 30 cm. Single square fields of 4 x 4 cm^2^, 10 x 10 cm^2^, and 15 x 15 cm^2^ field sizes were planned in Eclipse with the isocenter being at the center of the solid water. Each field was planned for 100 MU, using a beam energy of 6 MV. Additionally, each set of solid water was imaged twice: once lying flat on the table and once standing on its side. Anterior-posterior (AP) and posterior-anterior (PA) beams were planned with the solid water flat on the table. Lateral (LAT) beams were planned with the solid water on its side to exclude dose calculation effects caused by the treatment couch.

Each treatment plan was imported into EPIgray and a point of interest (POI) was manually defined at the beam isocenter and 1.5 cm away from the isocenter.

Multiple Fields and IMRT Plans

In a second step, six treatment plans of two or more beams were created based on the recommendations from the AAPM Task Group Report TG-119 [23]. These were planned and calculated in Eclipse. All beams used 6 MV beam energy. A list of these plans is summarized in Table 1. The plans were delivered to a block of solid water with the dimensions 15 x 30 x 30 cm^3^. The treatment couch was included in the dose calculation. Calculation points were at the beam isocenter and at additional points suggested by TG-119 [23]. All plans were also delivered to the ArcCHECK® (SunNuclear, Melbourne, FL) to establish a ground truth for delivery accuracy. The γ-index passing rate for 3%/3 mm criteria with a 10% dose threshold, which is the clinic’s standard for patient-specific IMRT quality assurance (QA), is also summarized in Table 1.

**Table 1:** Summary of the TG-119 test plans and results of the measured versus planned plan accuracy using the ArcCheck AP: anterior-posterior; PA: posterior-anterior; IMRT: intensity-modulated proton therapy; MU: monitor units

Plan	# of beams	Total MU	γ-index passing rate (3%/3 mm)
AP/PA	2	470	-
Bands	2	200	-
Multitarget	7	1,043	99.7
Prostate IMRT	7	1,452	100.0
Prostate IMRT (MU reduced by 5%)	7	1,379	98.3
Head and Neck IMRT	9	2,577	98.8
C-shape	9	1,846	99.3

**Figure 4:**
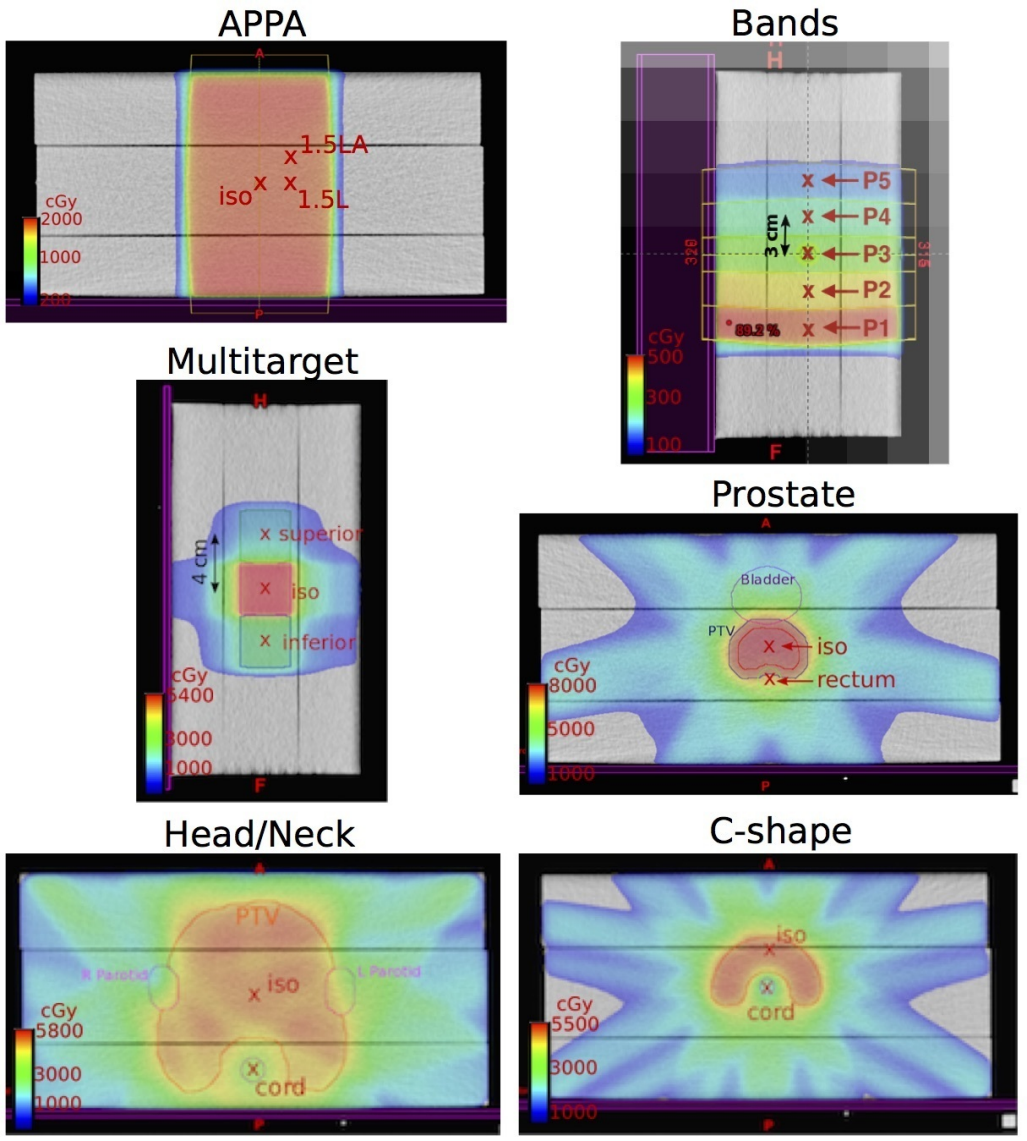
Color wash of the dose distribution for each plan The red x marks the evaluation points. AP: anterior-posterior; PA: posterior-anterior

Figure 4 shows the dose distribution of each plan and the location of the evaluation points.

System Sensitivity

The prostate plan was chosen to also test the sensitivity of the system. For that purpose, the prostate plan was copied and the MUs for each beam were reduced by 5%. The plan was delivered with the reduced number of MUs and the measurement was compared to the originally planned treatment.  

## Results

EPIgray software commissioning

Once the dosimetry mode on the Varian TrueBeam was calibrated, the data acquisition required for the software commissioning took about three hours of measurements per beam energy and per machine. The additional correction of the conversion factors reduced a systematic discrepancy between AP/PA and lateral beams for single fields by 2%, which was previously in the range of 3-3.5%. This added additional time to the overall duration of the commissioning process. EPIgray analyzes local dose differences.

Single fields

**Figure 5:**
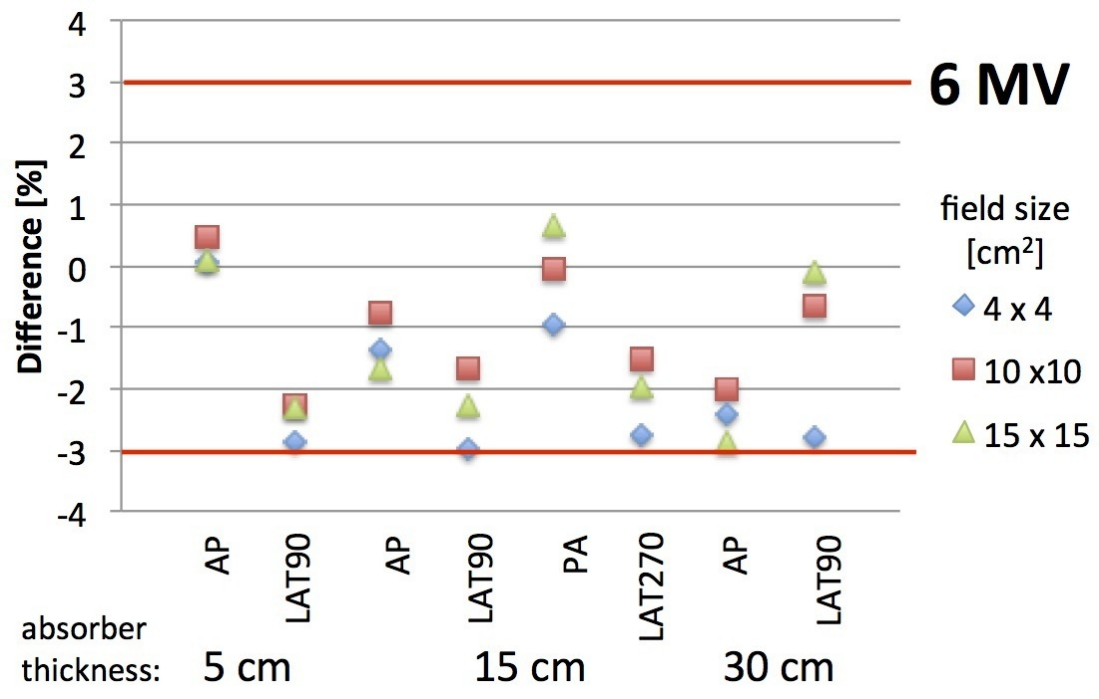
Percentage difference of reconstructed dose relative to the TPS dose for square fields with 6 MV beam energy. The field sizes are 4 x 4, 10 x 10, and 15 x 15 cm^2^. The thickness of the absorber is increased from 5 cm to 15 cm and 30 cm as indicated along the x-axis. TPS: treatment planning system; MV: megavoltage; AP: anterior-posterior; LAT: lateral

The delivery accuracy was expressed in percent difference per point and field. The results for anterior-posterior and lateral fields are summarized in Figure 5 for absorber thicknesses of 5 cm, 15 cm, and 30 cm. When the differences are negative, the reconstructed dose is smaller than the dose calculated by the TPS. For single square fields, the dose in EPIgray was reconstructed within 3% accuracy at the isocenter relative to the planned dose. The mean difference and standard deviation of all AP/PA fields was -0.9% ± 1.2 and of all lateral fields is -2.0% ± 0.9.

Multiple fields and IMRT plans

The relative deviation of the total dose was accurately reconstructed within 3% for all IMRT plans with points placed inside a high-dose region near the isocenter. Predictions became less accurate than 5% when the evaluation point was outside of the treatment target. Additionally, the dose at points 5 cm or more away from the isocenter or within an avoidance structure was calculated less reliably. For the IMRT plans, the average of the standard deviation for evaluation points inside an organ at risk (OAR) was 5.4%. The following paragraphs summarize the differences between the TPS dose and the reconstructed EPIgray dose for multiple-field plans and IMRT plans. Differences are shown for each beam and each calculation point in the figures below.

AP/PA

**Figure 6:**
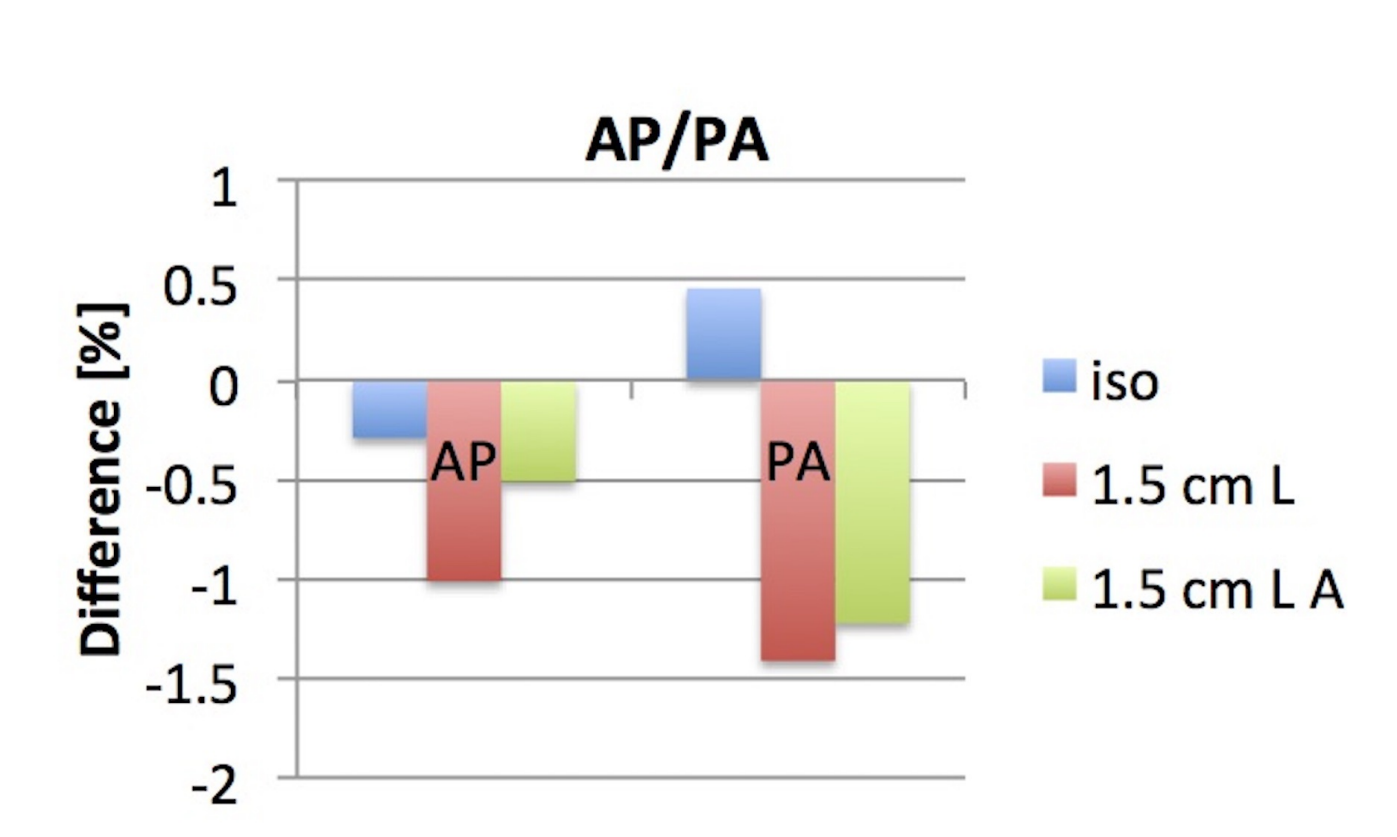
Difference in TPS and reconstructed dose for an AP/PA plan Dose difference evaluation at three different points: at isocenter, 1.5 cm left (L) of isocenter, and 1.5 cm left-anterior (LA) of isocenter. TPS: treatment planning system; AP: anterior-posterior; PA: posterior-anterior

The reconstructed dose of the AP/PA treatment with a 10 x 10 cm^2^ field size was within ± 0.5% of the TPS calculated dose at isocenter and within ± 1.5% for points 1.5 cm away from the  isocenter (Figure 6).

Bands

**Figure 7:**
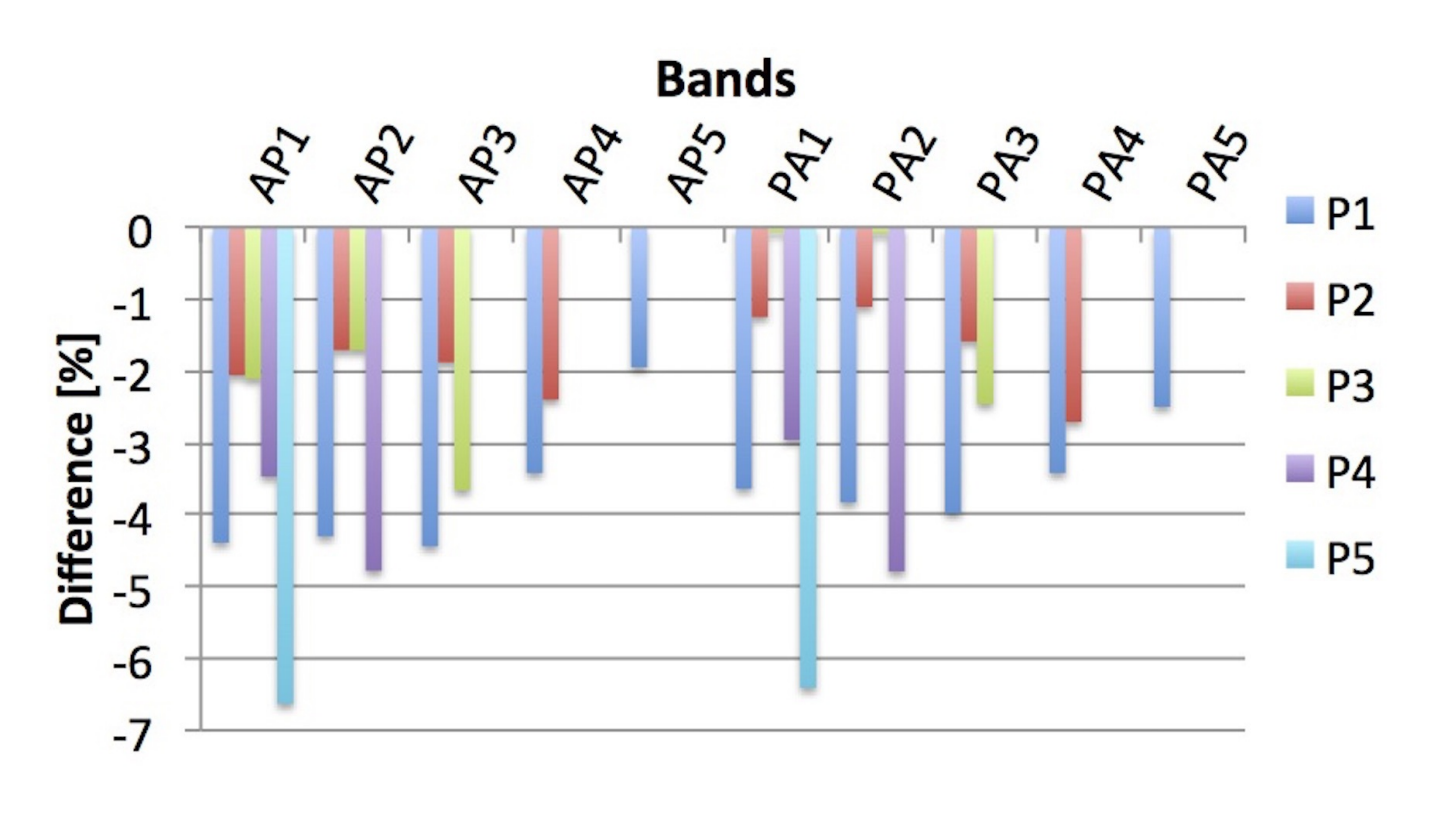
Difference in TPS and reconstructed dose for the plan ‘bands’ Points placed outside of the according treatment field were excluded. TPS: treatment planning system; AP: anterior-posterior; PA: posterior-anterior

The relative difference between the TPS dose and the reconstructed dose for the ‘bands’ treatment plan was within 7% for all five points when inside the treatment field (Figure 7). The mean difference of all fields and points was -3.0% with a standard deviation of 1.6%.

Multitarget

**Figure 8:**
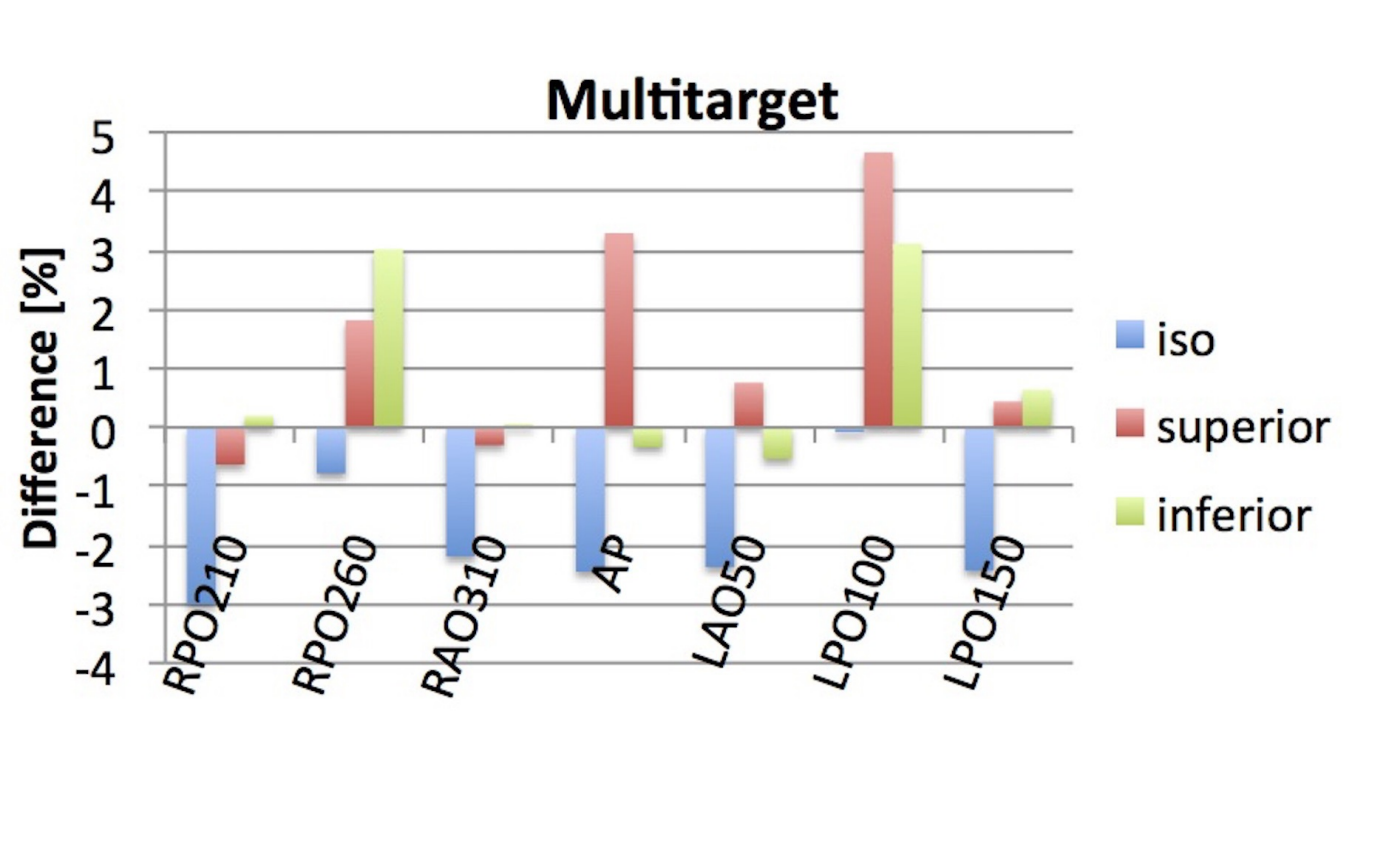
Difference in TPS and reconstructed dose for the plan ‘multitarget’. In addition to the isocenter point, points were also placed 2 cm superior and inferior to the isocenter. TPS: treatment planning system; AP: anterior-posterior; RPO: right posterior oblique, RAO: right anterior oblique, LAO: left anterior oblique, LPO: left posterior oblique

For the ‘multitarget’ plan, the γ-index passing rate for 3%/3 mm criteria was 99.7% with the ArcCheck in an independent plan-specific QA measurement. In EPIgray, the dose reconstruction for each beam and each point was within 5% of the planned dose. At the isocenter, the mean dose difference was -1.9% with a standard deviation of 1.1. For the superior and inferior points, the mean difference and standard deviation equaled 1.4% ± 1.9 and 0.9% ± 1.5, respectively (Figure 8).

Prostate

**Figure 9:**
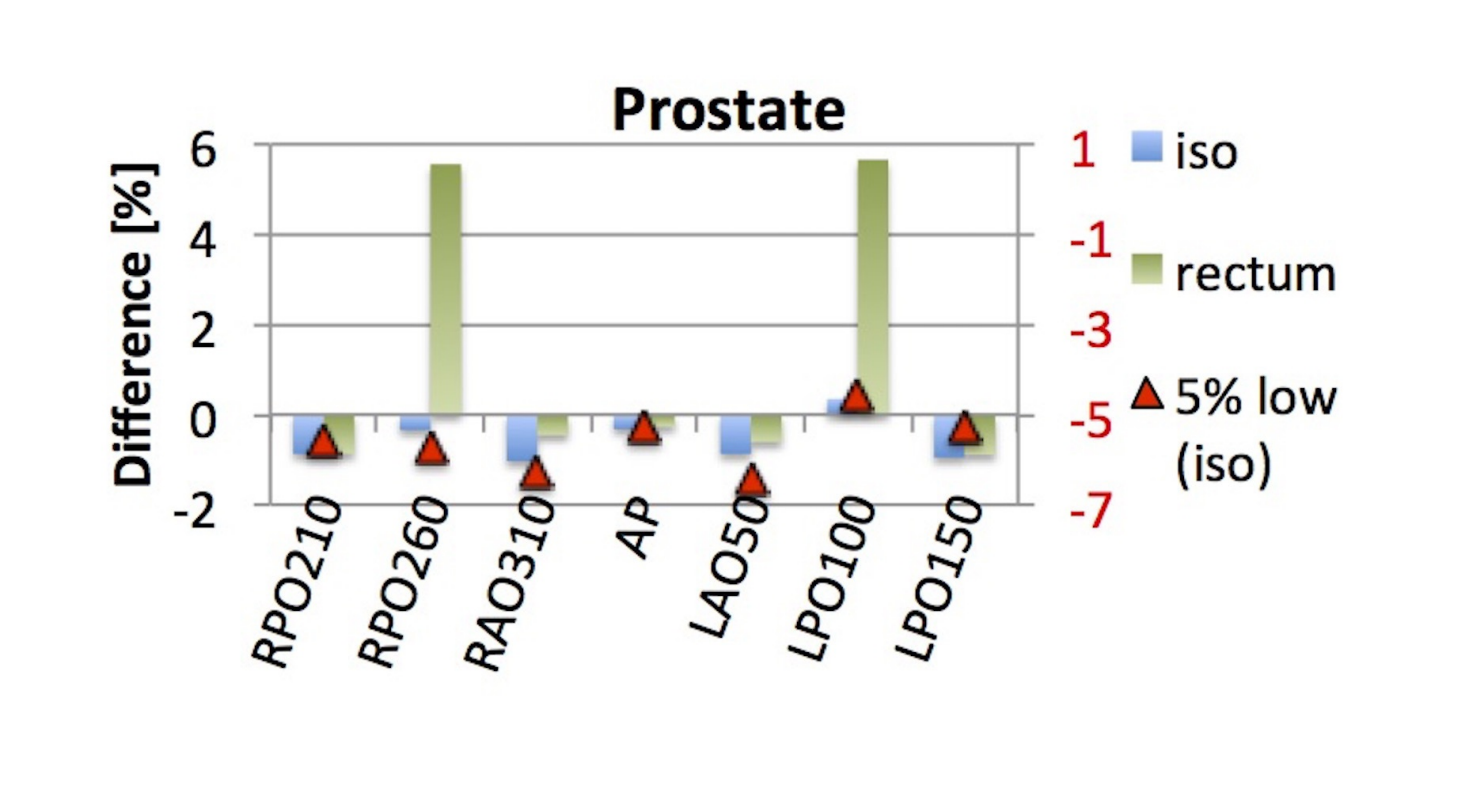
Difference in TPS and reconstructed dose for the IMRT prostate plan An additional point was placed 2 cm posterior to the isocenter. The red axis on the right shows the percentage difference for the prostate plan with the MU reduced by 5% (5% low, iso). TPS: treatment planning system; IMRT: intensity-modulated proton therapy; MU: monitor units; AP: anterior-posterior; RPO: right posterior oblique, RAO: right anterior oblique, LAO: left anterior oblique, LPO: left posterior oblique

The ArcCheck QA for the prostate plan yielded a γ-index passing rate of 100% for 3%/3 mm criteria. Figure 9 shows the percentage difference of  the TPS and reconstructed dose  for the IMRT prostate plan in blue  and green for points at the isocenter  and inside the rectum (avoidance structure), respectively. The evaluation points in the isocenter and the rectum were reconstructed within 1% for all beams, except RPO260 and LPO100. These two beams showed a 5.6% and 5.7% difference, respectively, in the reconstructed dose for the rectum calculation point. Both beams combined contributed < 10% of the total rectum dose to the evaluation point inside the rectum, which equals less than 50 cGy per fraction, suggesting that low-dose regions are not suitable as evaluation points. The mean difference and standard deviation was -0.6% ± 0.5 for the isocenter point and 1.16% ± 3.0 for the rectum point. The red axis on the right shows the percentage difference for the prostate plan, which was delivered with the MU reduced by 5%. In that case, the mean difference for the point at isocenter was -5.6% with a standard deviation of 0.6. Based on that, EPIgray reflected the 5% decrease in MU accurately. The γ-index for 3%/3 mm criteria was 98.3% for this plan.

Head and Neck

**Figure 10:**
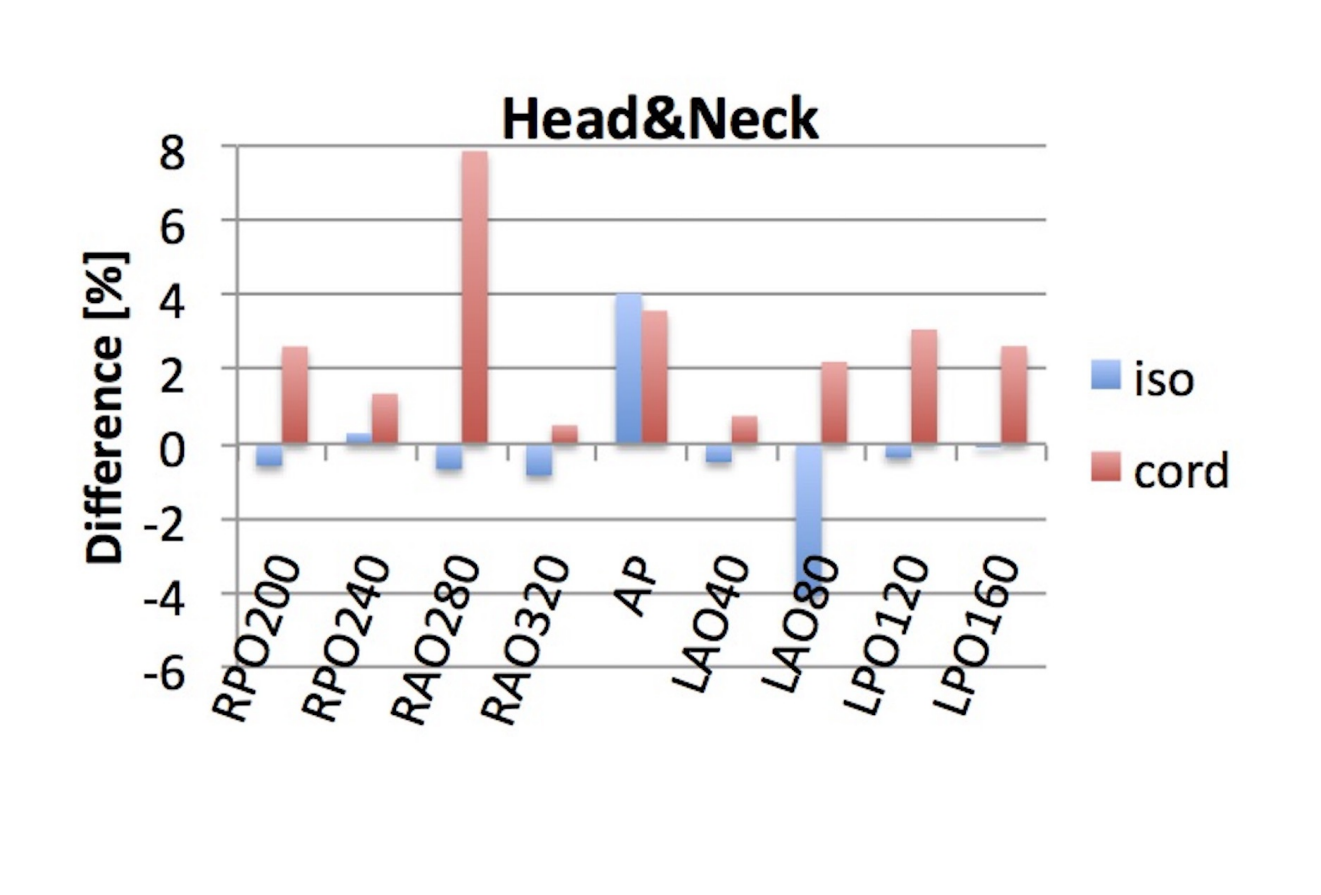
Difference in TPS and reconstructed dose for the IMRT Head and Neck plan An additional point was placed posterior to the isocenter inside the spinal cord. TPS: treatment planning system; IMRT: intensity-modulated proton therapy; AP: anterior-posterior; RPO: right posterior oblique, RAO: right anterior oblique, LAO: left anterior oblique, LPO: left posterior oblique

Figure 10 summarizes the difference in TPS and reconstructed dose for the IMRT head and neck plan. The γ-index passing rate for this plan was 98.8% for 3%/3mm criteria on the ArcCheck. In EPIgray, the reconstructed dose at the isocenter was  within 5% difference to the  planned dose for all beams. The dose  contribution from beam RAO280 to the cord was reconstructed 8% different to the planned dose, which corresponds to a 3 cGy absolute dose difference. The mean difference and standard deviation for the point at the isocenter and inside the cord was -0.3% ± 2.1 and 2.7% ± 2.2, respectively.

C-Shape

**Figure 11:**
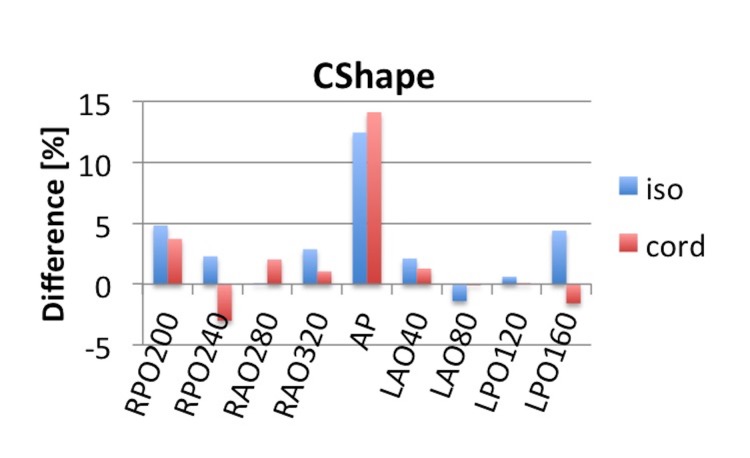
Difference in TPS and reconstructed dose for the IMRT CShape plan. The TPS and reconstructed dose is compared at isocenter and at a point inside the spinal cord. TPS: treatment planning system; IMRT: intensity-modulated proton therapy; AP: anterior-posterior; RPO: right posterior oblique, RAO: right anterior oblique, LAO: left anterior oblique, LPO: left posterior oblique

The ArcCheck QA measurement resulted in a γ-index of 99.3% for 3%/3 mm criteria. In EPIgray, the reconstructed and planned dose was  within 5% difference at the isocenter and within the cord except for  the dose delivered by the AP  beam, which was reconstructed  14.1% higher than the planned dose. To avoid the cord, this beam contributed very little dose to the isocenter and the cord. The 14.1% corresponds to a 2.4 cGy absolute dose difference in the reconstructed cord dose. The overall mean difference at the isocenter was 3.1% with a standard deviation of 4.0 and inside the cord was 2.0% ± 5.0. The results are presented in Figure 11.

## Discussion

The software commissioning measurements followed a simple process and the practical guide provided all the necessary information. However, in our case, the adjustment of the conversion factors showed an improvement in the results, particularly for lateral fields, and was done in addition to the instructions provided by DOSIsoft. The reason for the discrepancy in dose reconstruction between AP/PA and lateral beams remains under investigation. It is likely an effect caused by the modeling of the treatment couch.

A regular QA process for the validity of the model should be implemented. Thus, a monthly spot-check of the commissioning measurements should be considered. Validation measurements should also be performed after re-calibration of the EPID. 

The initial system setup was complicated by the combination of products by different vendors used for the linear accelerator and patient management system in our clinic. Thus, additional software was required to extract the portal images without modification to the DICOM header information from the treatment machine to the EPIgray system. This was unrelated to the EPIgray software.

The dose reconstruction for single fields slightly underestimated the actual delivered dose, particularly for lateral fields, which indicates a systematic error that has not been clearly identified. A similar underestimation was noticeable for the treatment plan ‘bands’. Based on our results, evaluation points in low-dose regions had lower passing rates than those in high-dose regions, likely because the former was excluded by the majority of control points per beam. In addition, EPIgray analyzes local dose differences, which are very sensitive to low doses. This may also explain some of the deviations seen for the IMRT plans, which use smaller segments than 3D plans. Furthermore, points outside the treatment field had insufficient passing rates. The plan ‘bands’ suggests that dose reconstruction is more accurate for evaluation points close to the treatment target center as well as in high-dose regions. This shows that not all evaluation points are equally suitable for evaluation. A new software version provides the option to use a set of 20 automatically generated points inside the prescription target volume (PTV), which are placed based on the DICOM RT_Dose file. The average dose difference of all points is provided for the full treatment fraction instead of individual beams and is thus statistically more significant. This could further reduce the deviations seen for IMRT plans. During this study, all points were chosen manually. The results suggest that a threshold of 5% may be appropriate to receive notifications of needed analysis and possible intervention. With additional experience and baseline performance of complex plans, it could be reasonable to increase the threshold up to 8% for individual beams.

For most IMRT plans, the reconstructed dose at the isocenter underestimates the planned dose by < 3%. Celi et al. found similar results in their study, in which they evaluated multiple treatment fractions of IMRT plans [22]. They found that the deviations between reconstructed and planned dose increase with the modulation of the plan. Consequently, discrepancies between IVD and the planned treatment delivery increase for more heterogeneous treatment sites.

An additional aspect worth noting is that EPIgray displays the relative deviation of the dose in the dose plan summary. However, this may not always be a good passing criterion without consideration of its standard deviation since individual points may still be outside the set threshold.

Overall, EPIgray provides a useful tool for EPID-based IVD that allows for inter-fractional dose monitoring for safe radiation delivery within 5% of the planned dose at the isocenter. It should be considered as a gross IVD check. However, it is not recommended as a tool that could replace patient-specific pre-treatment QA. The placement of evaluation points may largely influence whether or not the reconstructed dose is within a set threshold value. This issue should be resolved in the current updated software version, which automatically chooses 20 points within the PTV. Furthermore, while this study only evaluated 3D and IMRT plans, EPIgray is able to evaluate volumetric modulated arc radiotherapy (VMAT) deliveries in the updated version as well, which are also included in the study by Celi et al.

## Conclusions

The EPIgray formalism accuracy is adequate for an efficient error detection system. The software evaluates point dose measurements to verify the treatment plan delivery and patient positioning within 3-5% accuracy. However, the placement of evaluation points influences the dose reconstruction accuracy. Due to the local dose difference analysis, low-dose regions are less suitable as evaluation points. EPIgray provides immediate intra-fractional feedback on the delivery of treatment plans without affecting the treatment beam. Besides the EPID, it does not require additional hardware, which makes it accessible to all modern clinics. It is important to keep in mind that EPIgray is not intended to replace patient-specific quality assurance but should rather be utilized as an additional layer of safety for continuous patient treatment verification.
